# 2,2′-(Disulfanedi­yl)dibenzoic acid–2,9-dimethyl­phenanthroline–tetra­hydro­furan (1/2/1)

**DOI:** 10.1107/S1600536810037165

**Published:** 2010-09-25

**Authors:** Hadi D. Arman, Trupta Kaulgud, Edward R. T. Tiekink

**Affiliations:** a Department of Chemistry, The University of Texas at San Antonio, One UTSA Circle, San Antonio, Texas 78249-0698, USA; b Department of Chemistry, University of Malaya, 50603 Kuala Lumpur, Malaysia

## Abstract

The asymmetric unit of the title co-crystal solvate, C_14_H_10_O_4_S_2_·2C_14_H_12_N_2_·C_4_H_8_O, comprises a 2,2′-(disulfanedi­yl)dibenzoic acid mol­ecule, two mol­ecules of 2,9-dimethyl­phenanthroline and a tetra­hydro­furan (THF) solvent mol­ecule. Each end of the twisted diacid [dihedral angle between the benzene rings = 74.33 (17)°] forms a strong O—H⋯N hydrogen bond with a 2,9-dimethyl­phenanthroline mol­ecule, forming a trimeric aggregate. The crystal structure comprises layers of acid and THF mol­ecules, and layers of 2,9-dimethyl­phenanthroline mol­ecules that alternate along the *a* axis, the main connections between them being of the type C—H⋯O.

## Related literature

For related studies on co-crystal formation involving 2-[(2-carb­oxy­phen­yl)disulfan­yl]benzoic acid, see: Broker & Tiekink (2007 ▶, 2010 ▶); Broker *et al.* (2008 ▶). For a co-crystal involving 2,9-dimethyl­phenanthroline, see: Arman *et al.* (2010 ▶).
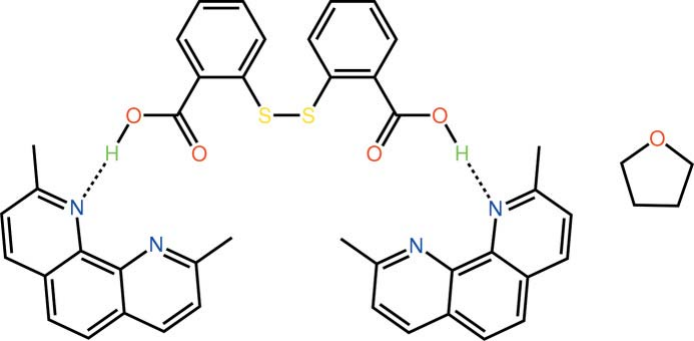


## Experimental

### 

#### Crystal data

C_14_H_10_O_4_S_2_·2C_14_H_12_N_2_·C_4_H_8_O
*M* *_r_* = 794.96
Monoclinic, 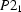

*a* = 14.011 (4) Å
*b* = 8.516 (3) Å
*c* = 17.403 (5) Å
β = 109.637 (6)°
*V* = 1955.7 (10) Å^3^
*Z* = 2
Mo *K*α radiation
μ = 0.19 mm^−1^
*T* = 98 K
0.26 × 0.21 × 0.10 mm

#### Data collection

Rigaku AFC12/SATURN724 diffractometer
13023 measured reflections
8637 independent reflections
7988 reflections with *I* > 2σ(*I*)
*R*_int_ = 0.048

#### Refinement

*R*[*F* ^2^ > 2σ(*F* ^2^)] = 0.068
*wR*(*F* ^2^) = 0.169
*S* = 1.05
8637 reflections
499 parameters
8 restraints
H atoms treated by a mixture of independent and constrained refinement
Δρ_max_ = 0.83 e Å^−3^
Δρ_min_ = −0.85 e Å^−3^
Absolute structure: Flack (1983 ▶), 3550 Friedel pairs
Flack parameter: 0.01 (9)

### 

Data collection: *CrystalClear* (Molecular Structure Corporation & Rigaku, 2005 ▶); cell refinement: *CrystalClear*; data reduction: *CrystalClear*; program(s) used to solve structure: *SHELXS97* (Sheldrick, 2008 ▶); program(s) used to refine structure: *SHELXL97* (Sheldrick, 2008 ▶); molecular graphics: *ORTEP-3* (Farrugia, 1997 ▶) and *DIAMOND* (Brandenburg, 2006 ▶); software used to prepare material for publication: *publCIF* (Westrip, 2010 ▶).

## Supplementary Material

Crystal structure: contains datablocks global, I. DOI: 10.1107/S1600536810037165/su2213sup1.cif

Structure factors: contains datablocks I. DOI: 10.1107/S1600536810037165/su2213Isup2.hkl

Additional supplementary materials:  crystallographic information; 3D view; checkCIF report

## Figures and Tables

**Table 1:** Hydrogen-bond geometry (Å, °)

*D*—H⋯*A*	*D*—H	H⋯*A*	*D*⋯*A*	*D*—H⋯*A*
O2—H2*o*⋯N1^i^	0.85 (4)	1.91 (4)	2.734 (4)	163 (4)
O2—H2*o*⋯N2^i^	0.85 (4)	2.46 (4)	2.982 (5)	121 (4)
O4—H4*o*⋯N4^ii^	0.84 (4)	1.86 (3)	2.691 (4)	170 (4)
C19—H19⋯O1^iii^	0.95	2.59	3.448 (5)	150
C22—H22⋯O5	0.95	2.52	3.383 (7)	150
C23—H23⋯O3	0.95	2.56	3.345 (5)	140

Symmetry codes: (i) 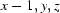; (ii) 
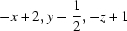
; (iii) 
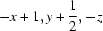
.

**Fig. 1.**
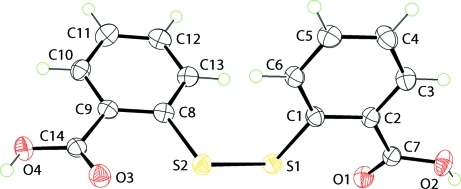
Molecular structure of 2-[(2-carboxyphenyl)disulfanyl]benzoic acid found in the title co-crystal solvate, showing the atom-labelling scheme and displacement ellipsoids at the 50% probability level.

**Fig. 2.**
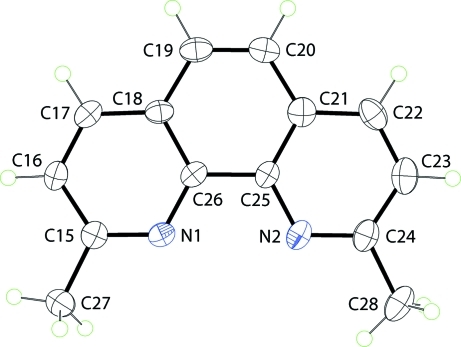
Molecular structure of first independent molecule of 2,9-dimethylphenanthroline found in the title co-crystal solvate, showing the atom-labelling scheme and displacement ellipsoids at the 50% probability level.

**Fig. 3.**
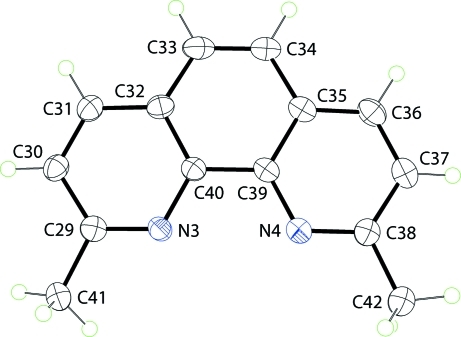
Molecular structure of second independent molecule of 2,9-dimethylphenanthroline found in the title co-crystal solvate, showing the atom-labelling scheme and displacement ellipsoids at the 50% probability level.

**Fig. 4.**
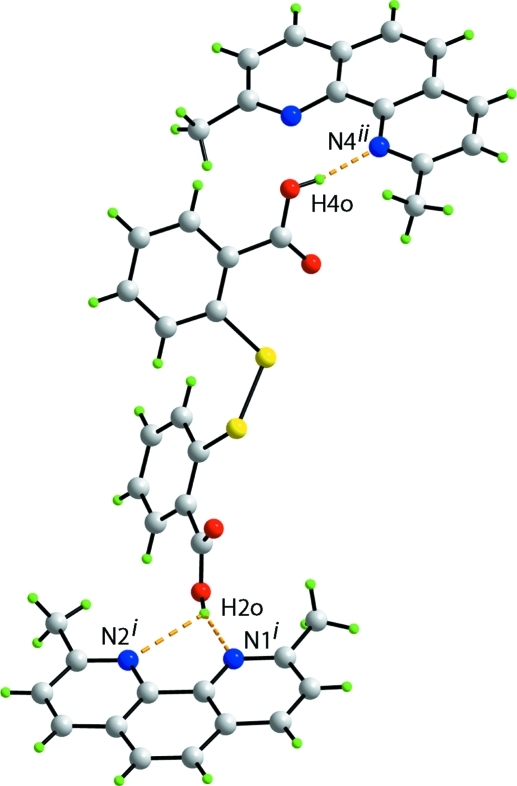
The trimeric aggregate, comprising a molecule of 2-[(2-carboxyphenyl)disulfanyl]benzoic acid and the two independent molecules of 2,9-dimethylphenanthroline, sustained by O—H···N hydrogen bonds (dashed lines) in the structure of the title co-crystal solvate [see Table 1 for details].

**Fig. 5.**
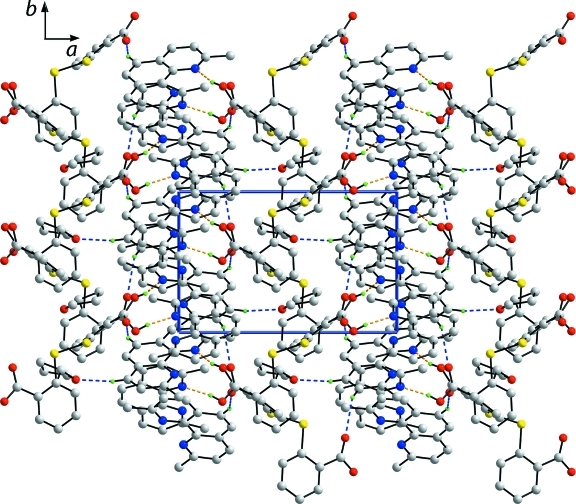
Stacking of alternating layers along the a axis in the title co-crystal solvate. The O—H···N hydrogen bonding and C—H···O interactions are shown as orange and blue dashed lines, respectively [see Table 1 for details. Hydrogen atoms not involved in intermolecular interactions have been removed for reasons of clarity].

## supplementary crystallographic information

### Comment

As a continuation of studies into the phenomenon of co-crystallization of 2-[(2-carboxyphenyl)disulfanyl]benzoic acid (Broker & Tiekink, 2007; Broker *et al.*, 2008; Broker & Tiekink, 2010; Arman *et al.*, 2010), the co-crystallization of this dithiodibenzoic acid and 2,9-dimethylphenanthroline was investigated. This lead to the isolation of the title co-crystal Tetrahydrofuran (thf) solvate.

The crystallographic asymmetric unit of the title compound comprises one molecule of dithiodibenzoic acid (Fig. 1), two molecules of 2,9-dimethylphenanthroline (Figs. 2 and 3), and a solvent thf molecule. The acid adopts the expected conformation (Broker & Tiekink, 2007), stabilized in part by two close *S*···*O*(carbonyl) interactions, *i.e*. S1···O1 = 2.713 (3) Å and S2···O3 = 2.711 (3) Å; the dihedral angle formed between the benzene rings = 74.33 (17) °. Each carboxylic acid-H forms a close hydrogen bond to a phenanthroline-N (Table 1), and in the case of the N1-phenanthroline molecule, a weaker O2—H···N2 interaction is noted; the equivalent O4—H···N4 contact is longer than 2.66 Å. These interactions result in the formation of a trimeric aggregate, Fig. 4.

In the crystal packing the dithiodibenzoic acid and tetrahydrofuran molecules assemble into layers in the *bc* plane interspersed by layers of 2,9-dimethylphenanthroline molecules, with the most prominent interactions between them being of the type C—H···O (see Fig. 5 and Table 1).

### Experimental

Gold coloured crystals of the title compound were obtained by the co-crystallization of 2-[(2-carboxyphenyl)disulfanyl]benzoic acid (Fluka, 0.02 mmol) and 2,9-dimethylphenanthroline (ACROS, 0.02 mmol) in tetrahydrofuran. Crystals were obtained by slow evaporation.

### Refinement

The O-bound H-atoms were located in a difference Fourier map and were refined with a distance restraint of O–H 0.84 (1) Å, and with *U*_iso_(H) = 1.5*U*_eq_(O). C-bound H-atoms were placed in calculated positions and were included in the refinement in the riding model approximation: C–H 0.95, 0.99 and 0.98 Å for CH, CH_2_ and CH_3_ H-atoms, respectively, with *U*_iso_(H) = k × *U*_eq_(C), where k = 1.5 for CH_3_ H-atoms and 1.2 for all other H-atoms. High thermal motion was associated with the tetrahydrofuran molecule but an alternate conformation could not be resolved. The constituent atoms were refined isotropically with O—C and C—C distance restraints of 1.43 (1) and 1.50 (1) Å, respectively. In the final refinement a low angle reflection evidently effected by the beam stop was omitted, *i.e*. (101).

### Crystal data

**Table:**

C_14_H_10_O_4_S_2_·2C_14_H_12_N_2_·C_4_H_8_O	*F*(000) = 836
*M**_r_* = 794.96	*D*_x_ = 1.350 Mg m^−^^3^
Monoclinic, *P*2_1_	Mo *K*α radiation, λ = 0.71073 Å
Hall symbol: P 2yb	Cell parameters from 8113 reflections
*a* = 14.011 (4) Å	θ = 2.3–40.2°
*b* = 8.516 (3) Å	µ = 0.19 mm^−^^1^
*c* = 17.403 (5) Å	*T* = 98 K
β = 109.637 (6)°	Block, gold
*V* = 1955.7 (10) Å^3^	0.26 × 0.21 × 0.10 mm
*Z* = 2	

### Data collection

**Table:**

Rigaku AFC12K/SATURN724 diffractometer	7988 reflections with *I* > 2σ(*I*)
Radiation source: fine-focus sealed tube	*R*_int_ = 0.048
graphite	θ_max_ = 27.5°, θ_min_ = 2.3°
ω scans	*h* = −15→18
13023 measured reflections	*k* = −10→11
8637 independent reflections	*l* = −22→21

### Refinement

**Table:**

Refinement on *F*^2^	Secondary atom site location: difference Fourier map
Least-squares matrix: full	Hydrogen site location: inferred from neighbouring sites
*R*[*F*^2^ > 2σ(*F*^2^)] = 0.068	H atoms treated by a mixture of independent and constrained refinement
*wR*(*F*^2^) = 0.169	*w* = 1/[σ^2^(*F*_o_^2^) + (0.0751*P*)^2^ + 1.2802*P*] where *P* = (*F*_o_^2^ + 2*F*_c_^2^)/3
*S* = 1.05	(Δ/σ)_max_ < 0.001
8637 reflections	Δρ_max_ = 0.83 e Å^−^^3^
499 parameters	Δρ_min_ = −0.85 e Å^−^^3^
8 restraints	Absolute structure: Flack (1983), 3550 Friedel pairs
Primary atom site location: structure-invariant direct methods	Flack parameter: 0.01 (9)

### Special details

**Table:**

Geometry. All s.u.'s (except the s.u. in the dihedral angle between two l.s. planes) are estimated using the full covariance matrix. The cell s.u.'s are taken into account individually in the estimation of s.u.'s in distances, angles and torsion angles; correlations between s.u.'s in cell parameters are only used when they are defined by crystal symmetry. An approximate (isotropic) treatment of cell s.u.'s is used for estimating s.u.'s involving l.s. planes.
Refinement. Refinement of *F*^2^ against ALL reflections. The weighted *R*-factor *wR* and goodness of fit *S* are based on *F*^2^, conventional *R*-factors *R* are based on *F*, with *F* set to zero for negative *F*^2^. The threshold expression of *F*^2^ > 2σ(*F*^2^) is used only for calculating *R*-factors(gt) *etc*. and is not relevant to the choice of reflections for refinement. *R*-factors based on *F*^2^ are statistically about twice as large as those based on *F*, and *R*- factors based on ALL data will be even larger.

### Fractional atomic coordinates and isotropic or equivalent isotropic displacement parameters (Å^2^)

**Table:**

	*x*	*y*	*z*	*U*_iso_*/*U*_eq_	
S1	0.42365 (6)	0.42502 (11)	0.23785 (5)	0.02513 (19)	
S2	0.57129 (6)	0.34896 (10)	0.26918 (6)	0.02557 (19)	
O1	0.24189 (19)	0.5734 (3)	0.19397 (16)	0.0292 (6)	
O2	0.2027 (2)	0.7481 (3)	0.27555 (17)	0.0333 (6)	
H2o	0.154 (3)	0.776 (6)	0.234 (2)	0.050*	
O3	0.76279 (19)	0.2507 (3)	0.29142 (16)	0.0289 (6)	
O4	0.80075 (19)	0.0087 (3)	0.25967 (17)	0.0283 (6)	
H4o	0.855 (2)	0.053 (5)	0.261 (3)	0.042*	
C1	0.4064 (2)	0.4616 (4)	0.3339 (2)	0.0239 (7)	
C2	0.3269 (2)	0.5589 (4)	0.3380 (2)	0.0230 (7)	
C3	0.3127 (3)	0.5853 (5)	0.4125 (2)	0.0279 (8)	
H3	0.2585	0.6502	0.4146	0.034*	
C4	0.3771 (3)	0.5176 (5)	0.4835 (2)	0.0314 (8)	
H4	0.3673	0.5359	0.5342	0.038*	
C5	0.4560 (3)	0.4227 (5)	0.4797 (2)	0.0294 (8)	
H5	0.4998	0.3750	0.5280	0.035*	
C6	0.4715 (2)	0.3967 (4)	0.4064 (2)	0.0242 (7)	
H6	0.5271	0.3340	0.4052	0.029*	
C7	0.2529 (2)	0.6278 (4)	0.2614 (2)	0.0233 (7)	
C8	0.5633 (3)	0.1399 (4)	0.2802 (2)	0.0243 (7)	
C9	0.6461 (3)	0.0442 (4)	0.2825 (2)	0.0228 (7)	
C10	0.6392 (3)	−0.1175 (4)	0.2914 (2)	0.0273 (8)	
H10	0.6940	−0.1827	0.2909	0.033*	
C11	0.5540 (3)	−0.1847 (5)	0.3008 (2)	0.0323 (8)	
H11	0.5516	−0.2946	0.3094	0.039*	
C12	0.4721 (3)	−0.0905 (5)	0.2977 (2)	0.0308 (8)	
H12	0.4127	−0.1363	0.3028	0.037*	
C13	0.4766 (3)	0.0702 (4)	0.2872 (2)	0.0272 (8)	
H13	0.4199	0.1337	0.2847	0.033*	
C14	0.7423 (3)	0.1115 (4)	0.2779 (2)	0.0236 (7)	
N1	1.0745 (2)	0.8741 (3)	0.13399 (18)	0.0244 (6)	
N2	0.9787 (3)	0.7029 (4)	0.21862 (19)	0.0282 (7)	
C15	1.1222 (3)	0.9556 (4)	0.0924 (2)	0.0276 (8)	
C16	1.0706 (3)	1.0298 (4)	0.0182 (2)	0.0280 (7)	
H16	1.1070	1.0878	−0.0095	0.034*	
C17	0.9668 (3)	1.0182 (4)	−0.0142 (2)	0.0289 (8)	
H17	0.9311	1.0682	−0.0644	0.035*	
C18	0.9138 (3)	0.9315 (4)	0.0276 (2)	0.0260 (7)	
C19	0.8062 (3)	0.9126 (5)	−0.0028 (2)	0.0313 (8)	
H19	0.7678	0.9592	−0.0533	0.038*	
C20	0.7588 (3)	0.8293 (5)	0.0396 (2)	0.0323 (8)	
H20	0.6873	0.8180	0.0182	0.039*	
C21	0.8140 (3)	0.7573 (5)	0.1164 (2)	0.0311 (8)	
C22	0.7672 (3)	0.6662 (5)	0.1615 (3)	0.0338 (9)	
H22	0.6958	0.6518	0.1424	0.041*	
C23	0.8265 (3)	0.5986 (5)	0.2336 (3)	0.0360 (9)	
H23	0.7962	0.5384	0.2653	0.043*	
C24	0.9327 (3)	0.6194 (4)	0.2602 (2)	0.0309 (8)	
C25	0.9211 (3)	0.7715 (4)	0.1483 (2)	0.0239 (7)	
C26	0.9722 (3)	0.8614 (4)	0.1026 (2)	0.0242 (7)	
C27	1.2360 (3)	0.9665 (5)	0.1295 (3)	0.0362 (9)	
H27A	1.2548	0.9921	0.1876	0.054*	
H27B	1.2610	1.0488	0.1017	0.054*	
H27C	1.2663	0.8656	0.1233	0.054*	
C28	0.9995 (4)	0.5427 (5)	0.3367 (3)	0.0404 (10)	
H28A	1.0702	0.5712	0.3459	0.061*	
H28B	0.9919	0.4284	0.3313	0.061*	
H28C	0.9801	0.5781	0.3829	0.061*	
N3	1.0152 (2)	0.4436 (4)	0.59852 (18)	0.0247 (6)	
N4	1.0154 (2)	0.6163 (4)	0.73176 (19)	0.0246 (6)	
C29	1.0161 (3)	0.3580 (5)	0.5358 (2)	0.0269 (7)	
C30	0.9254 (3)	0.3064 (4)	0.4751 (2)	0.0274 (7)	
H30	0.9281	0.2410	0.4316	0.033*	
C31	0.8345 (3)	0.3520 (5)	0.4802 (2)	0.0284 (7)	
H31	0.7734	0.3204	0.4393	0.034*	
C32	0.8309 (3)	0.4456 (4)	0.5455 (2)	0.0266 (7)	
C33	0.7390 (3)	0.5060 (5)	0.5519 (3)	0.0345 (9)	
H33	0.6766	0.4803	0.5109	0.041*	
C34	0.7386 (3)	0.5989 (5)	0.6146 (2)	0.0306 (8)	
H34	0.6761	0.6389	0.6166	0.037*	
C35	0.8316 (3)	0.6379 (4)	0.6785 (2)	0.0256 (7)	
C36	0.8348 (3)	0.7342 (4)	0.7439 (2)	0.0304 (8)	
H36	0.7739	0.7763	0.7481	0.036*	
C37	0.9266 (3)	0.7683 (5)	0.8025 (2)	0.0309 (8)	
H37	0.9294	0.8323	0.8479	0.037*	
C38	1.0165 (3)	0.7075 (4)	0.7948 (2)	0.0263 (7)	
C39	0.9249 (3)	0.5806 (4)	0.6737 (2)	0.0234 (7)	
C40	0.9247 (3)	0.4860 (4)	0.6051 (2)	0.0229 (7)	
C41	1.1178 (3)	0.3183 (5)	0.5301 (3)	0.0333 (9)	
H41A	1.1660	0.4023	0.5552	0.050*	
H41B	1.1119	0.3074	0.4726	0.050*	
H41C	1.1420	0.2192	0.5587	0.050*	
C42	1.1181 (3)	0.7423 (5)	0.8571 (2)	0.0313 (8)	
H42A	1.1562	0.6443	0.8734	0.047*	
H42B	1.1088	0.7915	0.9050	0.047*	
H42C	1.1557	0.8138	0.8336	0.047*	
O5	0.5266 (4)	0.6646 (6)	0.0293 (3)	0.0881 (15)*	
C43	0.4873 (6)	0.6959 (12)	−0.0568 (5)	0.106 (3)*	
H43A	0.5205	0.7896	−0.0701	0.127*	
H43B	0.4994	0.6053	−0.0879	0.127*	
C44	0.3809 (5)	0.7227 (10)	−0.0764 (4)	0.089 (2)*	
H44A	0.3429	0.6232	−0.0927	0.106*	
H44B	0.3563	0.7982	−0.1221	0.106*	
C45	0.3658 (5)	0.7886 (8)	−0.0006 (4)	0.0757 (18)*	
H45A	0.3626	0.9047	−0.0023	0.091*	
H45B	0.3034	0.7468	0.0064	0.091*	
C46	0.4594 (5)	0.7317 (10)	0.0663 (4)	0.086 (2)*	
H46A	0.4928	0.8205	0.1018	0.103*	
H46B	0.4406	0.6521	0.1001	0.103*	

### Atomic displacement parameters (Å^2^)

**Table:**

	*U*^11^	*U*^22^	*U*^33^	*U*^12^	*U*^13^	*U*^23^
S1	0.0238 (4)	0.0282 (4)	0.0225 (4)	0.0022 (3)	0.0066 (3)	0.0010 (3)
S2	0.0232 (4)	0.0245 (4)	0.0300 (5)	0.0000 (3)	0.0103 (3)	0.0003 (3)
O1	0.0279 (13)	0.0325 (14)	0.0231 (13)	0.0051 (11)	0.0031 (10)	0.0004 (11)
O2	0.0331 (14)	0.0318 (14)	0.0282 (14)	0.0131 (12)	0.0013 (10)	−0.0029 (12)
O3	0.0247 (13)	0.0295 (13)	0.0332 (14)	−0.0019 (11)	0.0105 (10)	−0.0017 (11)
O4	0.0208 (12)	0.0308 (14)	0.0338 (15)	0.0012 (10)	0.0100 (11)	−0.0037 (11)
C1	0.0205 (15)	0.0267 (17)	0.0247 (18)	0.0008 (13)	0.0078 (13)	−0.0005 (13)
C2	0.0181 (15)	0.0241 (17)	0.0249 (17)	0.0001 (12)	0.0049 (12)	−0.0004 (13)
C3	0.0241 (17)	0.0323 (19)	0.0251 (18)	0.0027 (15)	0.0053 (13)	−0.0032 (15)
C4	0.0263 (18)	0.044 (2)	0.0239 (19)	0.0037 (16)	0.0084 (14)	−0.0019 (16)
C5	0.0250 (17)	0.0366 (19)	0.0228 (17)	−0.0004 (15)	0.0029 (13)	0.0014 (16)
C6	0.0162 (14)	0.0308 (19)	0.0239 (17)	0.0011 (12)	0.0043 (12)	0.0010 (13)
C7	0.0193 (15)	0.0226 (16)	0.0272 (18)	−0.0007 (13)	0.0068 (13)	−0.0005 (14)
C8	0.0256 (17)	0.0248 (17)	0.0214 (17)	−0.0037 (13)	0.0063 (13)	−0.0030 (13)
C9	0.0228 (16)	0.0272 (17)	0.0168 (16)	−0.0022 (13)	0.0045 (12)	−0.0016 (13)
C10	0.0261 (17)	0.0273 (19)	0.0257 (18)	0.0025 (13)	0.0048 (13)	0.0003 (13)
C11	0.0299 (19)	0.0268 (19)	0.035 (2)	−0.0048 (14)	0.0034 (15)	−0.0009 (15)
C12	0.0277 (17)	0.0354 (19)	0.0283 (18)	−0.0086 (15)	0.0083 (14)	−0.0029 (16)
C13	0.0217 (16)	0.0313 (19)	0.0252 (18)	−0.0025 (14)	0.0035 (13)	0.0005 (14)
C14	0.0224 (16)	0.0287 (18)	0.0191 (16)	0.0014 (13)	0.0062 (12)	0.0014 (13)
N1	0.0277 (14)	0.0212 (14)	0.0230 (15)	0.0033 (11)	0.0067 (11)	−0.0009 (11)
N2	0.0391 (18)	0.0235 (15)	0.0240 (16)	0.0011 (13)	0.0134 (13)	0.0011 (12)
C15	0.0317 (19)	0.0226 (17)	0.0290 (19)	0.0001 (14)	0.0109 (14)	−0.0020 (14)
C16	0.0353 (19)	0.0247 (17)	0.0277 (18)	0.0009 (14)	0.0154 (15)	0.0027 (14)
C17	0.038 (2)	0.0249 (18)	0.0220 (18)	0.0016 (15)	0.0077 (15)	0.0010 (14)
C18	0.0264 (17)	0.0234 (17)	0.0243 (17)	0.0017 (14)	0.0035 (13)	−0.0030 (14)
C19	0.0314 (19)	0.0303 (18)	0.0266 (18)	0.0046 (16)	0.0026 (14)	−0.0062 (16)
C20	0.0268 (18)	0.033 (2)	0.033 (2)	0.0011 (15)	0.0049 (14)	−0.0031 (16)
C21	0.035 (2)	0.0244 (17)	0.036 (2)	0.0039 (15)	0.0147 (15)	−0.0015 (15)
C22	0.030 (2)	0.0266 (19)	0.048 (2)	−0.0044 (15)	0.0171 (17)	−0.0074 (17)
C23	0.050 (2)	0.029 (2)	0.039 (2)	−0.0038 (18)	0.0269 (19)	−0.0044 (17)
C24	0.044 (2)	0.0213 (17)	0.031 (2)	0.0006 (15)	0.0175 (16)	−0.0005 (15)
C25	0.0318 (18)	0.0192 (16)	0.0221 (17)	0.0008 (13)	0.0108 (13)	−0.0007 (13)
C26	0.0314 (17)	0.0207 (15)	0.0204 (16)	0.0041 (14)	0.0086 (13)	−0.0019 (13)
C27	0.0285 (19)	0.039 (2)	0.040 (2)	0.0000 (16)	0.0105 (16)	0.0030 (17)
C28	0.062 (3)	0.035 (2)	0.026 (2)	0.001 (2)	0.0163 (19)	0.0050 (17)
N3	0.0235 (14)	0.0261 (15)	0.0256 (15)	0.0019 (12)	0.0099 (11)	−0.0006 (12)
N4	0.0258 (14)	0.0224 (14)	0.0271 (15)	0.0008 (11)	0.0111 (11)	0.0022 (12)
C29	0.0271 (17)	0.0280 (17)	0.0263 (18)	0.0003 (15)	0.0097 (13)	0.0024 (15)
C30	0.0296 (18)	0.0293 (18)	0.0236 (18)	−0.0045 (14)	0.0092 (14)	−0.0013 (14)
C31	0.0284 (17)	0.0299 (17)	0.0253 (18)	−0.0022 (15)	0.0068 (13)	−0.0008 (15)
C32	0.0244 (16)	0.0262 (18)	0.0286 (18)	−0.0032 (14)	0.0081 (13)	0.0023 (14)
C33	0.0212 (18)	0.044 (2)	0.036 (2)	−0.0018 (16)	0.0069 (15)	0.0039 (18)
C34	0.0222 (17)	0.0341 (19)	0.035 (2)	−0.0004 (15)	0.0086 (14)	0.0037 (16)
C35	0.0261 (17)	0.0228 (16)	0.0296 (19)	0.0042 (13)	0.0118 (14)	0.0078 (14)
C36	0.0304 (19)	0.0273 (18)	0.036 (2)	0.0088 (15)	0.0144 (15)	0.0049 (16)
C37	0.037 (2)	0.0285 (19)	0.031 (2)	0.0044 (16)	0.0167 (15)	0.0021 (15)
C38	0.0301 (18)	0.0248 (17)	0.0248 (18)	−0.0017 (14)	0.0103 (14)	0.0029 (14)
C39	0.0242 (16)	0.0200 (16)	0.0285 (18)	0.0017 (13)	0.0120 (13)	0.0038 (13)
C40	0.0234 (16)	0.0211 (16)	0.0257 (18)	−0.0009 (13)	0.0101 (13)	0.0020 (13)
C41	0.0319 (19)	0.037 (2)	0.032 (2)	0.0035 (16)	0.0118 (15)	−0.0070 (16)
C42	0.034 (2)	0.0302 (18)	0.029 (2)	−0.0022 (16)	0.0102 (15)	−0.0015 (16)

### Geometric parameters (Å, °)

**Table:**

S1—C1	1.795 (4)	C23—H23	0.9500
S1—S2	2.0586 (13)	C24—C28	1.496 (6)
S2—C8	1.798 (4)	C25—C26	1.455 (5)
O1—C7	1.222 (4)	C27—H27A	0.9800
O2—C7	1.312 (4)	C27—H27B	0.9800
O2—H2o	0.85 (4)	C27—H27C	0.9800
O3—C14	1.224 (5)	C28—H28A	0.9800
O4—C14	1.309 (4)	C28—H28B	0.9800
O4—H4o	0.84 (4)	C28—H28C	0.9800
C1—C6	1.398 (5)	N3—C29	1.316 (5)
C1—C2	1.409 (5)	N3—C40	1.360 (4)
C2—C3	1.395 (5)	N4—C38	1.340 (5)
C2—C7	1.506 (5)	N4—C39	1.365 (4)
C3—C4	1.387 (5)	C29—C30	1.423 (5)
C3—H3	0.9500	C29—C41	1.500 (5)
C4—C5	1.389 (5)	C30—C31	1.362 (5)
C4—H4	0.9500	C30—H30	0.9500
C5—C6	1.382 (5)	C31—C32	1.404 (5)
C5—H5	0.9500	C31—H31	0.9500
C6—H6	0.9500	C32—C40	1.417 (5)
C8—C13	1.393 (5)	C32—C33	1.424 (5)
C8—C9	1.408 (5)	C33—C34	1.351 (6)
C9—C10	1.392 (5)	C33—H33	0.9500
C9—C14	1.490 (5)	C34—C35	1.439 (5)
C10—C11	1.383 (5)	C34—H34	0.9500
C10—H10	0.9500	C35—C36	1.391 (5)
C11—C12	1.386 (6)	C35—C39	1.425 (5)
C11—H11	0.9500	C36—C37	1.378 (6)
C12—C13	1.385 (6)	C36—H36	0.9500
C12—H12	0.9500	C37—C38	1.409 (5)
C13—H13	0.9500	C37—H37	0.9500
N1—C15	1.333 (5)	C38—C42	1.502 (5)
N1—C26	1.357 (4)	C39—C40	1.439 (5)
N2—C24	1.326 (5)	C41—H41A	0.9800
N2—C25	1.351 (5)	C41—H41B	0.9800
C15—C16	1.401 (5)	C41—H41C	0.9800
C15—C27	1.509 (5)	C42—H42A	0.9800
C16—C17	1.375 (5)	C42—H42B	0.9800
C16—H16	0.9500	C42—H42C	0.9800
C17—C18	1.411 (5)	O5—C46	1.426 (7)
C17—H17	0.9500	O5—C43	1.436 (7)
C18—C26	1.417 (5)	C43—C44	1.432 (7)
C18—C19	1.429 (5)	C43—H43A	0.9900
C19—C20	1.348 (6)	C43—H43B	0.9900
C19—H19	0.9500	C44—C45	1.512 (7)
C20—C21	1.437 (5)	C44—H44A	0.9900
C20—H20	0.9500	C44—H44B	0.9900
C21—C22	1.413 (6)	C45—C46	1.511 (7)
C21—C25	1.419 (5)	C45—H45A	0.9900
C22—C23	1.377 (6)	C45—H45B	0.9900
C22—H22	0.9500	C46—H46A	0.9900
C23—C24	1.413 (6)	C46—H46B	0.9900
			
C1—S1—S2	104.22 (12)	H27A—C27—H27B	109.5
C8—S2—S1	104.28 (13)	C15—C27—H27C	109.5
C7—O2—H2O	113 (4)	H27A—C27—H27C	109.5
C14—O4—H4O	109 (3)	H27B—C27—H27C	109.5
C6—C1—C2	118.3 (3)	C24—C28—H28A	109.5
C6—C1—S1	121.3 (3)	C24—C28—H28B	109.5
C2—C1—S1	120.3 (3)	H28A—C28—H28B	109.5
C3—C2—C1	120.3 (3)	C24—C28—H28C	109.5
C3—C2—C7	119.3 (3)	H28A—C28—H28C	109.5
C1—C2—C7	120.4 (3)	H28B—C28—H28C	109.5
C4—C3—C2	120.5 (3)	C29—N3—C40	119.1 (3)
C4—C3—H3	119.7	C38—N4—C39	119.2 (3)
C2—C3—H3	119.7	N3—C29—C30	122.1 (3)
C3—C4—C5	119.3 (4)	N3—C29—C41	117.0 (3)
C3—C4—H4	120.4	C30—C29—C41	120.8 (3)
C5—C4—H4	120.4	C31—C30—C29	119.0 (4)
C6—C5—C4	120.8 (3)	C31—C30—H30	120.5
C6—C5—H5	119.6	C29—C30—H30	120.5
C4—C5—H5	119.6	C30—C31—C32	120.3 (3)
C5—C6—C1	120.8 (3)	C30—C31—H31	119.9
C5—C6—H6	119.6	C32—C31—H31	119.9
C1—C6—H6	119.6	C31—C32—C40	116.9 (3)
O1—C7—O2	125.0 (3)	C31—C32—C33	123.2 (3)
O1—C7—C2	122.0 (3)	C40—C32—C33	119.8 (4)
O2—C7—C2	113.0 (3)	C34—C33—C32	121.6 (4)
C13—C8—C9	119.1 (3)	C34—C33—H33	119.2
C13—C8—S2	121.2 (3)	C32—C33—H33	119.2
C9—C8—S2	119.7 (3)	C33—C34—C35	120.7 (4)
C10—C9—C8	119.2 (3)	C33—C34—H34	119.6
C10—C9—C14	118.9 (3)	C35—C34—H34	119.6
C8—C9—C14	121.9 (3)	C36—C35—C39	118.2 (3)
C11—C10—C9	121.2 (3)	C36—C35—C34	122.7 (3)
C11—C10—H10	119.4	C39—C35—C34	119.0 (4)
C9—C10—H10	119.4	C37—C36—C35	119.7 (4)
C10—C11—C12	119.5 (4)	C37—C36—H36	120.2
C10—C11—H11	120.3	C35—C36—H36	120.2
C12—C11—H11	120.3	C36—C37—C38	119.6 (4)
C13—C12—C11	120.2 (4)	C36—C37—H37	120.2
C13—C12—H12	119.9	C38—C37—H37	120.2
C11—C12—H12	119.9	N4—C38—C37	121.8 (3)
C12—C13—C8	120.8 (3)	N4—C38—C42	116.9 (3)
C12—C13—H13	119.6	C37—C38—C42	121.3 (4)
C8—C13—H13	119.6	N4—C39—C35	121.5 (3)
O3—C14—O4	124.6 (3)	N4—C39—C40	118.8 (3)
O3—C14—C9	121.3 (3)	C35—C39—C40	119.7 (3)
O4—C14—C9	114.0 (3)	N3—C40—C32	122.4 (3)
C15—N1—C26	118.5 (3)	N3—C40—C39	118.5 (3)
C24—N2—C25	118.3 (3)	C32—C40—C39	119.0 (3)
N1—C15—C16	122.5 (3)	C29—C41—H41A	109.5
N1—C15—C27	116.8 (3)	C29—C41—H41B	109.5
C16—C15—C27	120.8 (4)	H41A—C41—H41B	109.5
C17—C16—C15	119.6 (4)	C29—C41—H41C	109.5
C17—C16—H16	120.2	H41A—C41—H41C	109.5
C15—C16—H16	120.2	H41B—C41—H41C	109.5
C16—C17—C18	119.6 (3)	C38—C42—H42A	109.5
C16—C17—H17	120.2	C38—C42—H42B	109.5
C18—C17—H17	120.2	H42A—C42—H42B	109.5
C17—C18—C26	117.0 (3)	C38—C42—H42C	109.5
C17—C18—C19	122.7 (3)	H42A—C42—H42C	109.5
C26—C18—C19	120.3 (3)	H42B—C42—H42C	109.5
C20—C19—C18	120.6 (4)	C46—O5—C43	108.4 (6)
C20—C19—H19	119.7	C44—C43—O5	106.4 (6)
C18—C19—H19	119.7	C44—C43—H43A	110.5
C19—C20—C21	121.5 (4)	O5—C43—H43A	110.5
C19—C20—H20	119.2	C44—C43—H43B	110.5
C21—C20—H20	119.2	O5—C43—H43B	110.5
C22—C21—C25	117.1 (4)	H43A—C43—H43B	108.6
C22—C21—C20	123.1 (4)	C43—C44—C45	106.8 (6)
C25—C21—C20	119.7 (4)	C43—C44—H44A	110.4
C23—C22—C21	119.1 (4)	C45—C44—H44A	110.4
C23—C22—H22	120.5	C43—C44—H44B	110.4
C21—C22—H22	120.5	C45—C44—H44B	110.4
C22—C23—C24	119.4 (4)	H44A—C44—H44B	108.6
C22—C23—H23	120.3	C46—C45—C44	102.4 (6)
C24—C23—H23	120.3	C46—C45—H45A	111.3
N2—C24—C23	122.7 (4)	C44—C45—H45A	111.3
N2—C24—C28	116.5 (4)	C46—C45—H45B	111.3
C23—C24—C28	120.7 (4)	C44—C45—H45B	111.3
N2—C25—C21	123.4 (3)	H45A—C45—H45B	109.2
N2—C25—C26	118.0 (3)	O5—C46—C45	108.3 (6)
C21—C25—C26	118.7 (3)	O5—C46—H46A	110.0
N1—C26—C18	122.8 (3)	C45—C46—H46A	110.0
N1—C26—C25	118.0 (3)	O5—C46—H46B	110.0
C18—C26—C25	119.2 (3)	C45—C46—H46B	110.0
C15—C27—H27A	109.5	H46A—C46—H46B	108.4
C15—C27—H27B	109.5		
			
C1—S1—S2—C8	88.74 (17)	C24—N2—C25—C26	−179.3 (3)
S2—S1—C1—C6	−18.7 (3)	C22—C21—C25—N2	−0.1 (5)
S2—S1—C1—C2	160.5 (2)	C20—C21—C25—N2	−177.7 (3)
C6—C1—C2—C3	−1.8 (5)	C22—C21—C25—C26	178.6 (3)
S1—C1—C2—C3	179.0 (3)	C20—C21—C25—C26	1.0 (5)
C6—C1—C2—C7	−178.2 (3)	C15—N1—C26—C18	−0.2 (5)
S1—C1—C2—C7	2.6 (4)	C15—N1—C26—C25	179.7 (3)
C1—C2—C3—C4	0.7 (6)	C17—C18—C26—N1	−0.5 (5)
C7—C2—C3—C4	177.1 (3)	C19—C18—C26—N1	179.3 (3)
C2—C3—C4—C5	−0.1 (6)	C17—C18—C26—C25	179.7 (3)
C3—C4—C5—C6	0.7 (6)	C19—C18—C26—C25	−0.5 (5)
C4—C5—C6—C1	−1.9 (6)	N2—C25—C26—N1	−1.4 (5)
C2—C1—C6—C5	2.4 (5)	C21—C25—C26—N1	179.9 (3)
S1—C1—C6—C5	−178.4 (3)	N2—C25—C26—C18	178.5 (3)
C3—C2—C7—O1	−157.5 (4)	C21—C25—C26—C18	−0.2 (5)
C1—C2—C7—O1	19.0 (5)	C40—N3—C29—C30	−0.9 (5)
C3—C2—C7—O2	21.9 (5)	C40—N3—C29—C41	178.2 (3)
C1—C2—C7—O2	−161.7 (3)	N3—C29—C30—C31	2.7 (6)
S1—S2—C8—C13	−15.5 (3)	C41—C29—C30—C31	−176.5 (4)
S1—S2—C8—C9	165.0 (2)	C29—C30—C31—C32	−1.5 (6)
C13—C8—C9—C10	0.2 (5)	C30—C31—C32—C40	−1.1 (5)
S2—C8—C9—C10	179.7 (3)	C30—C31—C32—C33	175.7 (4)
C13—C8—C9—C14	−178.0 (3)	C31—C32—C33—C34	−178.3 (4)
S2—C8—C9—C14	1.4 (4)	C40—C32—C33—C34	−1.5 (6)
C8—C9—C10—C11	−2.3 (5)	C32—C33—C34—C35	−1.1 (6)
C14—C9—C10—C11	176.0 (3)	C33—C34—C35—C36	179.4 (4)
C9—C10—C11—C12	2.9 (6)	C33—C34—C35—C39	1.6 (6)
C10—C11—C12—C13	−1.6 (6)	C39—C35—C36—C37	−1.5 (5)
C11—C12—C13—C8	−0.5 (6)	C34—C35—C36—C37	−179.3 (4)
C9—C8—C13—C12	1.1 (5)	C35—C36—C37—C38	1.2 (6)
S2—C8—C13—C12	−178.4 (3)	C39—N4—C38—C37	−0.4 (5)
C10—C9—C14—O3	−160.7 (3)	C39—N4—C38—C42	179.7 (3)
C8—C9—C14—O3	17.6 (5)	C36—C37—C38—N4	−0.2 (6)
C10—C9—C14—O4	18.5 (5)	C36—C37—C38—C42	179.7 (4)
C8—C9—C14—O4	−163.2 (3)	C38—N4—C39—C35	0.0 (5)
C26—N1—C15—C16	0.7 (5)	C38—N4—C39—C40	178.4 (3)
C26—N1—C15—C27	−179.9 (3)	C36—C35—C39—N4	0.9 (5)
N1—C15—C16—C17	−0.6 (6)	C34—C35—C39—N4	178.8 (3)
C27—C15—C16—C17	−179.9 (4)	C36—C35—C39—C40	−177.4 (3)
C15—C16—C17—C18	−0.1 (5)	C34—C35—C39—C40	0.4 (5)
C16—C17—C18—C26	0.6 (5)	C29—N3—C40—C32	−1.9 (5)
C16—C17—C18—C19	−179.2 (4)	C29—N3—C40—C39	−179.5 (3)
C17—C18—C19—C20	−179.6 (4)	C31—C32—C40—N3	3.0 (5)
C26—C18—C19—C20	0.6 (6)	C33—C32—C40—N3	−174.0 (3)
C18—C19—C20—C21	0.2 (6)	C31—C32—C40—C39	−179.4 (3)
C19—C20—C21—C22	−178.5 (4)	C33—C32—C40—C39	3.6 (5)
C19—C20—C21—C25	−1.0 (6)	N4—C39—C40—N3	−3.7 (5)
C25—C21—C22—C23	0.9 (6)	C35—C39—C40—N3	174.7 (3)
C20—C21—C22—C23	178.5 (4)	N4—C39—C40—C32	178.6 (3)
C21—C22—C23—C24	−1.1 (6)	C35—C39—C40—C32	−3.0 (5)
C25—N2—C24—C23	0.4 (5)	C46—O5—C43—C44	−23.0 (9)
C25—N2—C24—C28	178.7 (3)	O5—C43—C44—C45	28.7 (9)
C22—C23—C24—N2	0.4 (6)	C43—C44—C45—C46	−22.9 (9)
C22—C23—C24—C28	−177.8 (4)	C43—O5—C46—C45	8.1 (9)
C24—N2—C25—C21	−0.6 (5)	C44—C45—C46—O5	8.9 (8)

### Hydrogen-bond geometry (Å, °)

**Table:**

*D*—H···*A*	*D*—H	H···*A*	*D*···*A*	*D*—H···*A*
O2—H2o···N1^i^	0.85 (4)	1.91 (4)	2.734 (4)	163 (4)
O2—H2o···N2^i^	0.85 (4)	2.46 (4)	2.982 (5)	121 (4)
O4—H4o···N4^ii^	0.84 (4)	1.86 (3)	2.691 (4)	170 (4)
C19—H19···O1^iii^	0.95	2.59	3.448 (5)	150
C22—H22···O5	0.95	2.52	3.383 (7)	150
C23—H23···O3	0.95	2.56	3.345 (5)	140

Symmetry codes: (i) *x*−1, *y*, *z*; (ii) −*x*+2, *y*−1/2, −*z*+1; (iii) −*x*+1, *y*+1/2, −*z*.
